# Tracheo-innominate artery fistula with continuous bleeding successfully treated through the suprasternal approach: a case report

**DOI:** 10.1186/s13019-020-1080-y

**Published:** 2020-02-24

**Authors:** Shotaro Kaneko, Keiji Uchida, Norihisa Karube, Keiichiro Kasama, Tomoyuki Minami, Tomoki Cho, Ryo Izubuchi, Kenichi Fushimi, Naoto Yabu, Motohiko Goda, Munetaka Masuda

**Affiliations:** 1 Cardiovascular Center, Yokohama City University Medical Center, 4-57 Urafune-cho, Minami-ku, Yokohama, 232-0024 Japan; 2 Department of Surgery, Yokohama City University Hospital, Yokohama, 236-0004 Japan

**Keywords:** Tracheo-innominate artery fistula, Continuous bleeding, Suprasternal approach, Balloon catheter

## Abstract

**Background:**

Tracheo-innominate artery fistula (TIF) is a rare but fatal complication occurring after tracheotomy. Brachiocephalic trunk transection, one of the surgical treatments for TIF, is mostly associated with a full or partial median sternotomy. We describe a case of TIF with continuous bleeding, which was successfully treated with brachiocephalic trunk transection through a collar incision without the need for median sternotomy.

**Case presentation:**

*Case 1.*

An 18-year-old man was referred to our hospital with bleeding from a tracheal stoma, which had ceased prior to admission. TIF was suspected after examination. Innominate artery transection was performed through a collar incision. TIF was not revealed when we cut the innominate artery anterior wall open; therefore, we opted for preventive surgical intervention. The post-operative course was uneventful, and the patient was asymptomatic at the 3-year follow-up.

*Case 2.*

A 14-year-old male patient was admitted to our hospital with bleeding from a tracheal stoma, and TIF was suspected after examination. There was persistent bleeding when the cuff of the tracheotomy tube was deflated. Brachiocephalic trunk transection was performed through a collar incision using balloon occlusion. The post-operative course was uneventful, and rebleeding has not occurred 2 years later.

**Conclusions:**

Brachiocephalic trunk transection without any median sternotomy may offer the benefits of post-operative infection prevention. In patients with suspected continuous bleeding, using a balloon catheter may be a safe and effective method of treatment.

## Background

Tracheo-innominate artery fistula (TIF) is a rare but fatal complication after tracheotomy, with an incidence varying from 0.1 to 1.0% [1]. Innominate artery transection, one of the surgical treatments in TIF, typically requires full or partial median sternotomy [2]. However, the surgical field is not aseptic in cases of tracheal fistula, which poses a risk of sternal osteomyelitis after sternotomy. As these patients often have severe neuromuscular disorders such as opisthotonos, which lead to increased mechanical pressure between the cervical spine and innominate vein, or have often undergone prolonged mechanical ventilation, less invasive surgical procedures are preferred. Here, we report two cases wherein the patients developed TIF and were successfully treated with brachiocephalic trunk transection without median sternotomy. Additionally, one of our patients had persistent bleeding and we created a bloodless operating field using balloon catheter occlusion.

## Case presentation

### Case 1

An 18-year-old man was referred to our hospital with bleeding from a tracheal stoma; the bleeding had ceased prior to admission. The patient had undergone tracheostomy due to hypoxic encephalopathy 4 years prior. Bronchoscopy showed an ulcer with the innominate artery at the base of the anterior surface of the trachea. Contrast-enhanced computed tomography (CECT) revealed the innominate artery to be in contact with the anterior surface of the trachea. TIF was suspected, and innominate artery transection was performed. The patient was placed in the supine position with the neck extended, and we made a cervical collar incision. We cut the platysma, anterior cervical muscle, and sternal head of right sternocleidomastoid muscle and exposed the right common carotid artery, right subclavian artery, and innominate artery, respectively. The TIF was not revealed when we cut the innominate artery anterior wall open; hence, we opted for preventive surgical intervention. Innominate artery transection was performed, and the stumps were closed with 5–0 polypropylene continuous suture. The post-operative course was uneventful, and we did not detect rebleeding, wound infection, and aneurysmal change of the aortic stump at the 3-year follow-up.

### Case 2

**Fig. 1:**
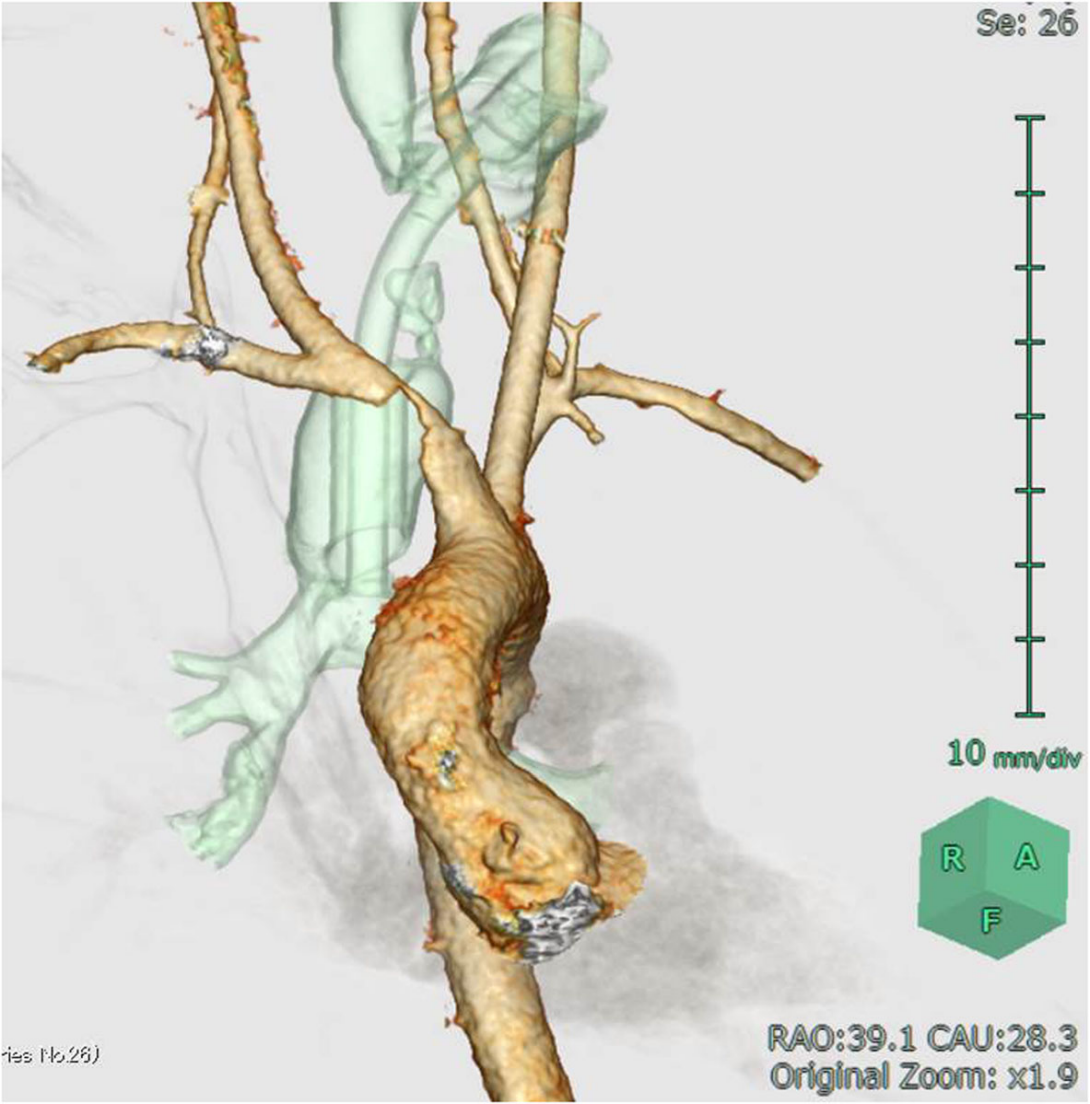
Pre-operative CECT in Case 2. Pre-operative CECT revealed that the innominate artery ran close to the anterior surface of the trachea. The part of the wall is irregular; therefore, it appeared to form a fistula in the site (CECT: contrast-enhanced computed tomography)

**Fig. 2:**
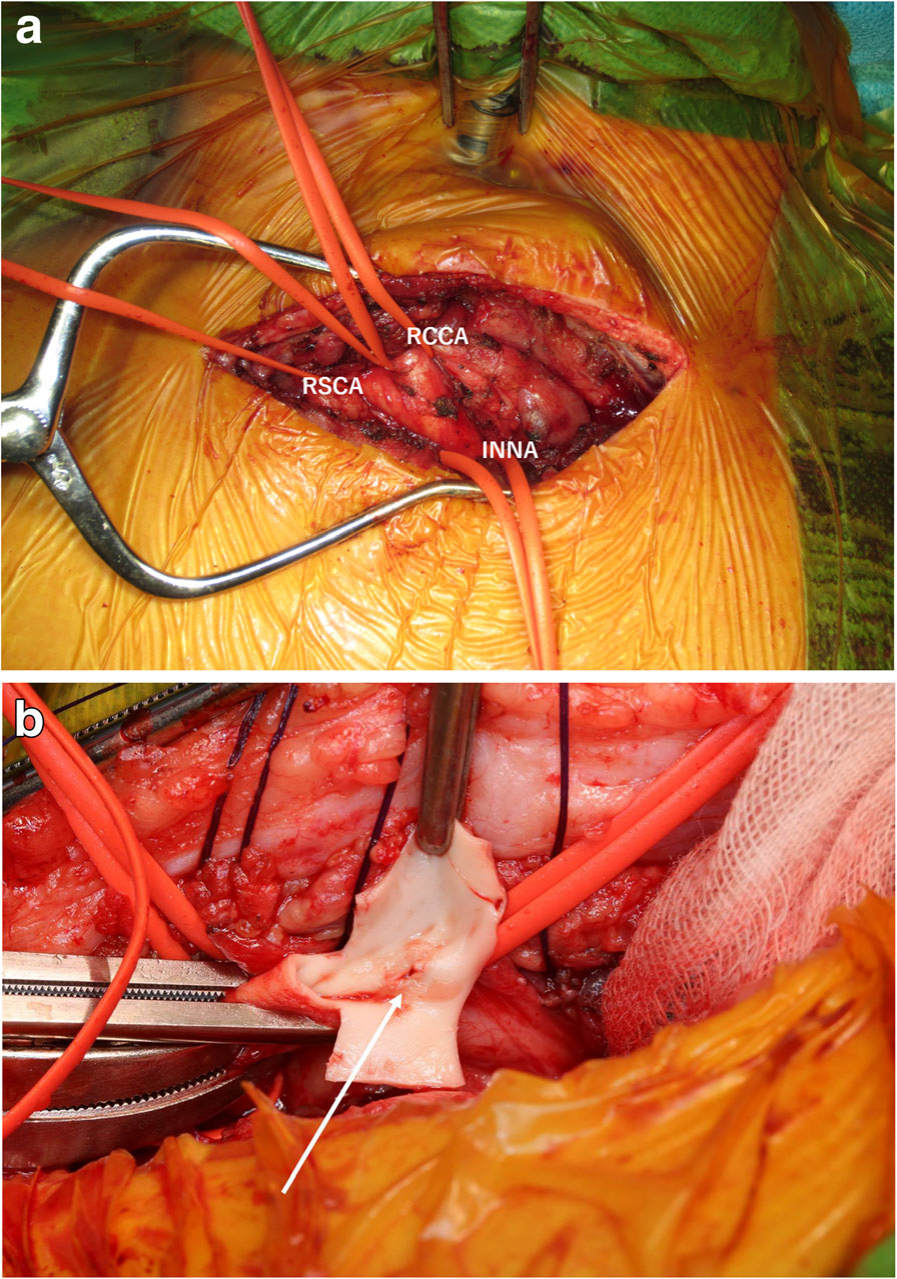
**a, b**: Operative views in Case 2. **a** We exposed the RCCA, RSCA, and INNA, respectively. **b** The TIF was revealed when we cut the anterior wall of the innominate artery (indicated by the white arrow) open (RCCA: right common carotid artery; RSCA: right subclavian artery; INNA: innominate artery)

**Fig. 3:**
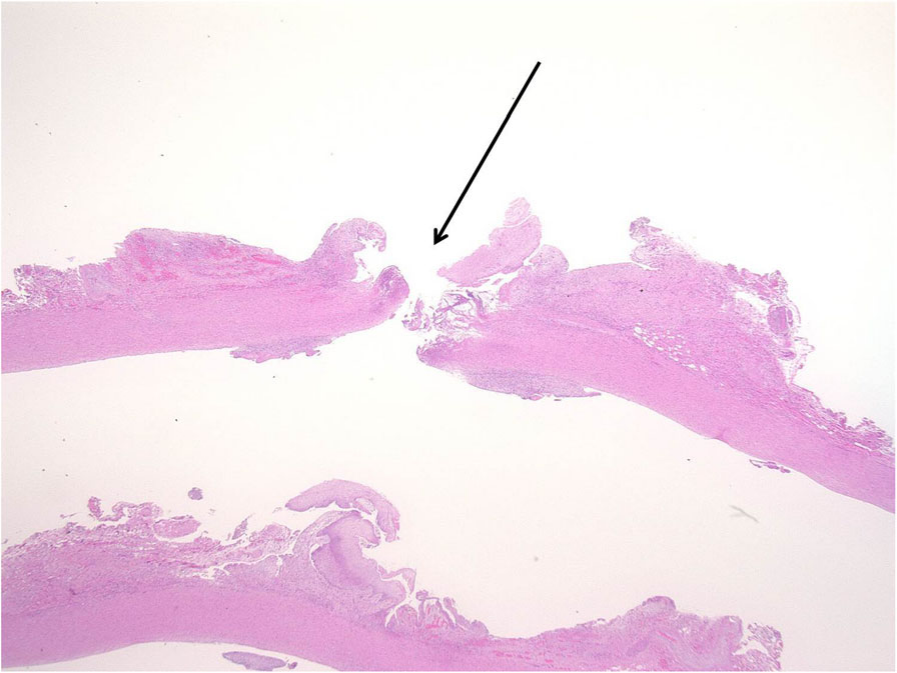
Neutrophilic infiltration and tracheal attachment to the adventitia in Case 2. Microscopic findings of the TIF (indicated by the black arrow) revealed intramural neutrophilic infiltration in the innominate artery and the attachment of tracheal tissue to adventitia. (stain, HE; magnification, × 10)

**Fig. 4:**
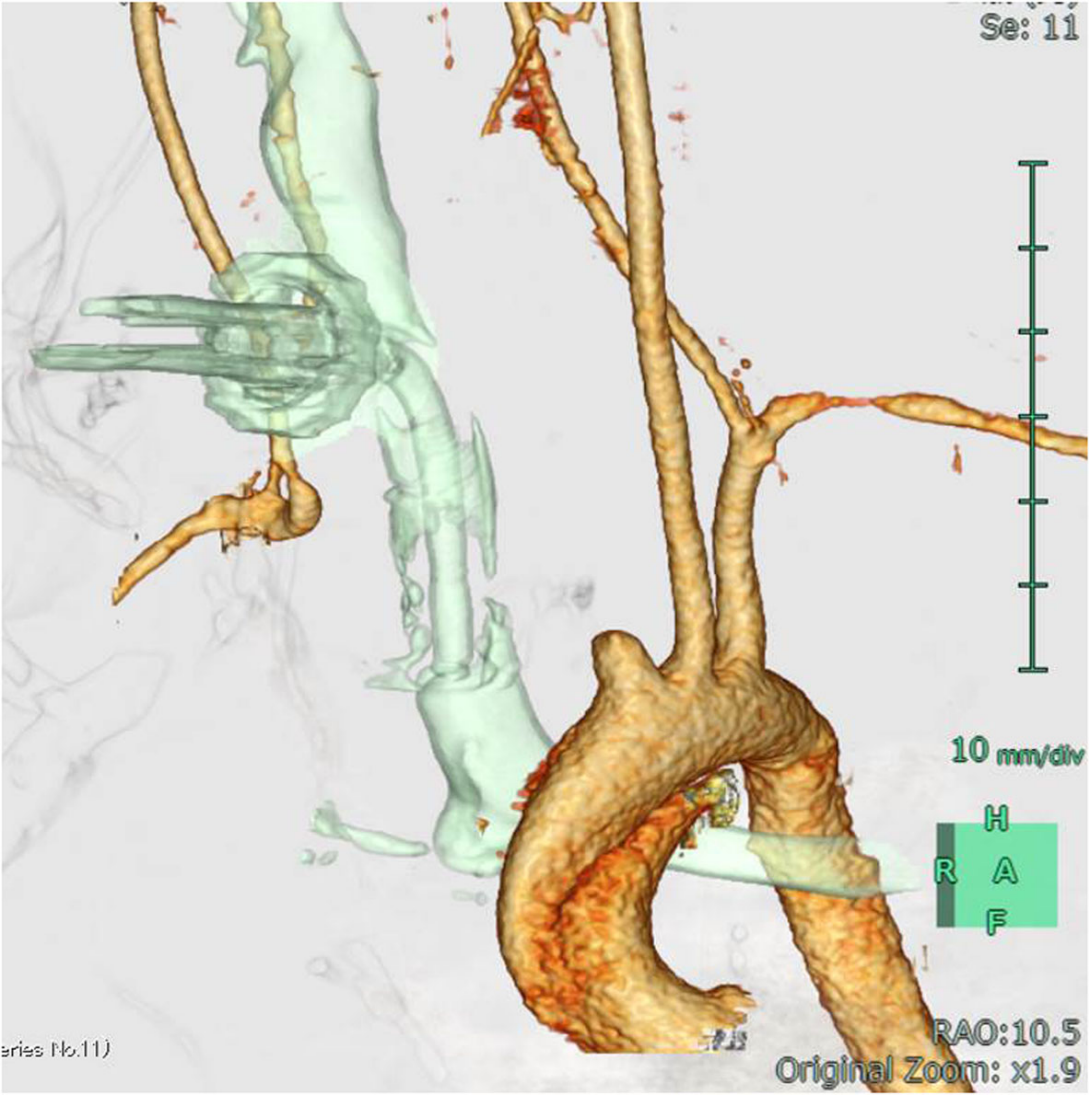
Post-operative CECT in Case 2. Post-operative CECT revealed the innominate artery, which was successfully transected (CECT: contrast-enhanced computed tomography)

A 14-year-old male patient was admitted to our hospital with bleeding from a tracheal stoma. The patient had undergone tracheostomy due to acute encephalopathy 12 years prior. On admission, temporary control of bleeding was achieved by overinflating the cuff of the tracheotomy tube. CECT revealed the innominate artery to be in contact with the anterior surface of the trachea (Fig. 1). TIF was suspected, and innominate artery transection was performed through a supra-sternal collar incision and exposed the right common carotid artery, right subclavian artery, and innominate artery in the same manner as in Case 1 (Fig. 2a). Due to the persistent bleeding when the cuff of the tracheotomy tube deflated, we inserted a 6-French Fogarty balloon catheter (Edwards Lifesciences, Irvine, CA, USA) from the distal side of the innominate artery to the aortic arch and expanded the balloon to create an occlusion. Balloon occlusion allowed for a bloodless field; thus, we were able to expose the proximal side of the innominate artery safely. After we sufficiently exposed the proximal side of the innominate artery, a cross clamp was simultaneously applied with balloon catheter withdrawal. We performed transection of the innominate artery, and the proximal and distal stumps were closed with 5–0 polypropylene continuous suture. Communication of the right common carotid artery and the right subclavian artery at the distal stump was maintained. The TIF was revealed when we cut the anterior wall of the innominate artery (Fig. 2b). Pathological analysis of the TIF revealed intramural neutrophilic infiltration in the innominate artery, and that the tracheal tissue was attached to the adventitia (Fig. 3). Post-operative CECT revealed that the innominate artery was transected and isolated from the site of tracheostomy (Fig. 4). The post-operative course was uneventful, and we did not detect rebleeding, wound infection, and aneurysmal change of the aortic stump more than 2 years after surgery.

## Discussion and conclusions

The management of patients with TIF includes endovascular stenting and surgical intervention. Although endovascular stenting is less invasive and simpler to perform, there is an increased risk of rebleeding and infection. Previous reports suggest that endovascular stenting should be used as a bridge to successful surgical intervention [1, 3], and we have no objection to it.

As previously mentioned, innominate artery transection typically requires a sternotomy. Fujimoto et al. advocated that innominate artery transection through the suprasternal approach is less invasive compared to previous partial or full sternotomy, and recommended preventive surgical intervention for the patients who are more likely to develop TIF [4]. The case that they reported was not due to continuous bleeding from the innominate artery. In our first case, bleeding was controlled pre-operatively, and the operation proceeded without any problems using the method proposed by Fujimoto et al. However, in cases with continuous bleeding, like case 2 in our study, fatal bleeding from TIF could occur; hence, we devised a safer operative technique utilizing balloon occlusion of the innominate artery.

When deciding on an appropriate procedure, CECT after hemostasis is the most important examination. Three-dimensional image reconstruction allows for the visualization of the positional relationship between the innominate artery and the sternum. If most of the innominate artery is located cranially, or at a level equal to that of the suprasternal notch, an approach without sternotomy can be selected. Furthermore, using a balloon catheter seems safe and effective in cases of suspected continuous bleeding. In our hospital, the surgical cases of TIF are only two cases that we demonstrated, and the accumulation of more cases is expected.

## Data Availability

The clinical dataset used in this case report are available from the corresponding author on reasonable request.

## Abbreviations

CECT: Contrast-enhanced computed tomography
TIF: Tracheo-innominate artery fistula
